# Selective Heart Irradiation Induces Cardiac Overexpression of the Pro-hypertrophic miR-212

**DOI:** 10.3389/fonc.2019.00598

**Published:** 2019-07-16

**Authors:** Márta Sárközy, Renáta Gáspár, Ágnes Zvara, Laura Kiscsatári, Zoltán Varga, Bence Kővári, Mónika G. Kovács, Gergő Szűcs, Gabriella Fábián, Petra Diószegi, Gábor Cserni, László G. Puskás, Thomas Thum, Zsuzsanna Kahán, Tamás Csont, Sándor Bátkai

**Affiliations:** ^1^Metabolic Diseases and Cell Signaling Group, Department of Biochemistry, Interdisciplinary Centre of Excellence, University of Szeged, Szeged, Hungary; ^2^Laboratory for Functional Genomics, Biological Research Center of the Hungarian Academy of Sciences, Institute of Genetics, Szeged, Hungary; ^3^Department of Oncotherapy, Faculty of Medicine, University of Szeged, Szeged, Hungary; ^4^Department of Pathology, University of Szeged, Szeged, Hungary; ^5^Institute of Molecular and Translational Therapeutic Strategies (IMTTS), Hanover Medical School, Hanover, Germany

**Keywords:** thoracic irradiation, left ventricular hypertrophy, heart failure with preserved ejection fraction (HFpEF), miRNA-212, FOXO3

## Abstract

**Background:** A deleterious, late-onset side effect of thoracic radiotherapy is the development of radiation-induced heart disease (RIHD). It covers a spectrum of cardiac pathology including also heart failure with preserved ejection fraction (HFpEF) characterized by left ventricular hypertrophy (LVH) and diastolic dysfunction. MicroRNA-212 (miR-212) is a crucial regulator of pathologic LVH via FOXO3-mediated pathways in pressure-overload-induced heart failure. We aimed to investigate whether miR-212 and its selected hypertrophy-associated targets play a role in the development of RIHD.

**Methods:** RIHD was induced by selective heart irradiation (50 Gy) in a clinically relevant rat model. One, three, and nineteen weeks after selective heart irradiation, transthoracic echocardiography was performed to monitor cardiac morphology and function. Cardiomyocyte hypertrophy and fibrosis were assessed by histology at week 19. qRT-PCR was performed to measure the gene expression changes of miR-212 and forkhead box O3 (FOXO3) in all follow-up time points. The cardiac transcript level of other selected hypertrophy-associated targets of miR-212 including extracellular signal-regulated kinase 2 (ERK2), myocyte enhancer factor 2a (MEF2a), AMP-activated protein kinase, (AMPK), heat shock protein 40 (HSP40), sirtuin 1, (SIRT1), calcineurin A-alpha and phosphatase and tensin homolog (PTEN) were also measured at week 19. Cardiac expression of FOXO3 and phospho-FOXO3 were investigated at the protein level by Western blot at week 19.

**Results:** In RIHD, diastolic dysfunction was present at every time point. Septal hypertrophy developed at week 3 and a marked LVH with interstitial fibrosis developed at week 19 in the irradiated hearts. In RIHD, cardiac miR-212 was overexpressed at week 3 and 19, and FOXO3 was repressed at the mRNA level only at week 19. In contrast, the total FOXO3 protein level failed to decrease in response to heart irradiation at week 19. Other selected hypertrophy-associated target genes failed to change at the mRNA level in RIHD at week 19.

**Conclusions:** LVH in RIHD was associated with cardiac overexpression of miR-212. However, miR-212 seems to play a role in the development of LVH via FOXO3-independent mechanisms in RIHD. As a central regulator of pathologic remodeling, miR-212 might become a novel target for RIHD-induced LVH and heart failure.

## Introduction

Radiotherapy has an important role, among other therapeutic modalities, in the treatment of thoracic tumors including breast, lung, esophageal and childhood cancers or Hodgkin's lymphoma. About half of cancer patients are treated with radiotherapy (1). Although the application of modern radiotherapy planning and delivery techniques significantly improves the radiation protection of the heart, in many cases, the whole heart, or part of it, still receives a dose sufficient enough to cause radiation-induced heart disease (RIHD) with chronic, and often severe consequences (1). RIHD develops many years or decades after radiotherapy in a dose-dependent manner (2, 3). Premature coronary heart disease, electrical conduct defects or valve abnormalities are often associated with RIHD (4–6). In the early phase, RIHD often presents as heart failure with preserved ejection fraction (HFpEF), characterized by LVH and diastolic dysfunction (1, 7, 8). Radiotherapy significantly improves cancer patient survival; however, in the long-term, patients are at risk of RIHD and subsequent heart failure which becomes a major health issue affecting outcome, quality of life and health care costs (1). Unfortunately, therapeutic options for RIHD are currently insufficient.

Radiation deteriorates the heart structure and function by inducing changes to the vasculature, i.e., coronary arteries and microvessels, and directly acting on the myocardium (9). The focus of the studies so far has been on the vascular effects, which indirectly influence the myocardium (3, 6, 10, 11). Recently, more data has become available on the radiation-induced direct effect on the myocardial structure. Interstitial inflammation and progressive fibrosis are well-known pathological effects of RIHD (12). They have been shown to be mediated by oxidative stress, the activation of pathologic NO-cGMP-PKG signaling, cytokine and growth factor cascades such as IL-4, IL-13, Rho/ROCK pathway and transforming growth factor beta (TGF-β) (1, 7, 9, 13, 14). Histopathological examination of cardiac lesions of RIHD shows hypertrophic cardiomyocytes, inflammatory cells, fibroblasts, and excessive deposition of collagens (1, 7, 14). Radiation-induced hypertrophy and fibrosis of the myocardium ultimately leads to a decrease in elasticity, ductility, and the development of diastolic heart failure. Only a few studies exist so far on the mechanisms of radiation-induced diffuse myocardial injury. The initial phase of RIHD includes reactive cardiac hypertrophy with enlarged cardiomyocytes (1, 7, 15). Very little is known about the mechanisms of cardiac hypertrophy and the pathological remodeling in RIHD. We have previously developed a clinically relevant rat model of RIHD, characterized by LVH and simultaneous interstitial fibrosis (16). Our present study is based on this RIHD model.

Endogenous microRNAs (miRs, 22 bp) are non-coding RNA species that are post-transcriptional regulators targeting specific mRNAs, resulting in an increase of mRNA degradation via complementary binding and the suppression of protein synthesis, thus influencing cellular function (17). miRs have been described as “master switches” in cardiovascular biology, and the dysregulation of specific miRs are key pathological factors in many cardiovascular diseases (18–21). The miR-212/132 cluster is considered to be a central regulator of the development of pressure-overload-induced LVH and heart failure via the repression of the anti-hypertrophic transcription factor FOXO3 in mice with transverse aortic constriction (TAC) (22). Moreover, the overexpression of miR-212 separate from miR-132, was reported to play a role in the development of LVH and heart failure via fetal gene reprogramming in human hearts (23). Furthermore, the pro-hypertrophic potential of miR-212 was also confirmed in primary neonatal rat cardiomyocytes (24). Beyond FOXO3, other predicted or validated LVH-associated direct targets of miR-212 have been identified. They include e.g., the extracellular signal-regulated kinase 2 (ERK2) (25), myocyte enhancer factor 2a (MEF2a) (26); AMP-activated protein kinase (AMPK) (27); heat shock protein 40 (HSP40) (28); sirtuin 1 (SIRT1) (29); calcineurin A-alpha (30); and phosphatase and tensin homolog (PTEN) (31).

So far there is no literature available on cardiac miR-212 and its targets in the development of RIHD. Therefore, we aimed to investigate the potential role of miR-212 and its selected hypertrophy-associated targets in the development of LVH during the early phases of RIHD.

## Materials and Methods

The datasets generated for this study are available on request from the corresponding author. This study was carried out in accordance with the recommendations of the National Institutes of Health Guide for the Care and Use of Laboratory Animals (NIH Publication No. 85-23, Revised 1996). The protocol was approved by the Animal Research Ethics Committee of Csongrád County (XV.1181/2013) and the University of Szeged. All institutional and national guidelines for the care and use of laboratory animals were followed.

### Animals

A total of 48 male Sprague-Dawley rats (200–220 g, 6–7 weeks old) were used (*n* = 8 in each group) in three separate experiments. After 1 week of acclimatization in a temperature-controlled room (22 ± 2°C; relative humidity 55 ± 10%), the animals were randomly assigned to different groups. A total of 24 animals received selective heart irradiation to induce RIHD, and a total of 24 animals served as controls. The animals were housed in pairs in individually ventilated cages (Sealsafe IVC system, Italy) in a temperature-controlled room with a 12 h:12 h light/dark cycle. Standard rat chow supplemented with 5% fat (Innovo Kft., Gödöllő, Hungary) and tap water were supplied *ad libitum* (16).

### Experimental Setup

**Figure 1:**
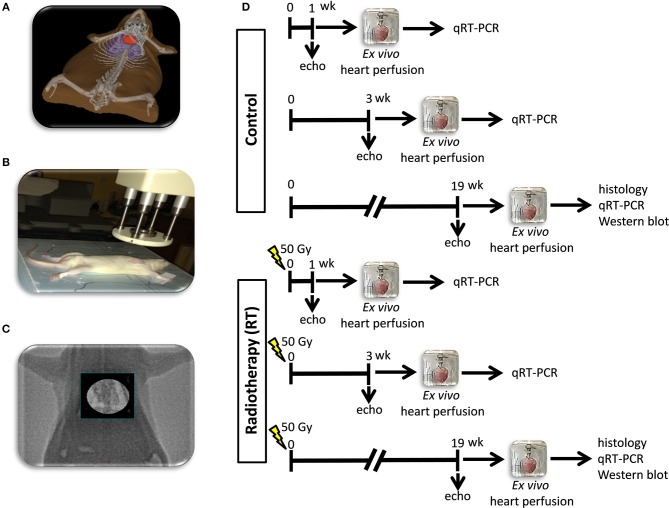
Experimental protocol figure. **(A)** 3D visualization of the rat heart based on CT scans, **(B)** positioning of the rat for delivering the radiation dose with a Primus linear accelerator under sodium pentobarbital anesthesia, **(C)** appropriate position of the animal proven by portal imaging, **(D)** experimental protocol.

Animals were divided into three control and three irradiated groups in separate experiments (*n* = 8 in each group) (Figure 1). In the irradiated groups, animals received a single dose of 50 Gy delivered to the whole heart to induce RIHD. Groups were followed-up for 1, 3, and 19 weeks, respectively. Cardiac morphology and function were assessed by transthoracic echocardiography in all time points. The development of LVH and fibrosis in chronic RIHD was verified by the measurement of myocardial fiber diameters as well as picrosirius red staining for collagen at week 19. Total RNA was isolated from the hearts, and the myocardial expression of miR-212 and its direct target FOXO3 were measured by qRT-PCR in every time point. Myocardial expression of selected mRNA targets beyond FOXO3 was also measured by qRT-PCR at week 19. Moreover, cardiac expression of total-FOXO3, phospho-FOXO3, total-AKT, and phospho-AKT were measured using Western blot technique at week 19.

### Heart Irradiation

Heart irradiation with a single dose of 50 Gy was carried out as described previously (16). Briefly, the planning of the irradiation was based on a 3D model (Figure 1A), and the dose was delivered to the geometric center of the heart. For better coverage of the heart and lung protection, a 6 MeV electron radiation was given with a circle-shaped aperture with a 2 cm diameter (Figure 1B). The radiation dose was delivered with a Primus linear accelerator (Siemens Healthcare GmbH, Erlangen, Germany) at a dose intensity of 5 Gy/min if the appropriate position of the animal was proven using a built-in electronic portal imaging device (Figure 1C). Before the irradiation, rats were anesthetized with sodium pentobarbital (Euthasol, ip. 40 mg/kg, Produlab Pharma b.v., Raamsdonksveer, The Netherlands), then fixed in the supine position to a flat surface couch.

### Transthoracic Echocardiography

Cardiac morphology and function were assessed by transthoracic echocardiography at week 1, 3, and 19 to monitor the development of RIHD (Figure 1D). Rats were anesthetized with 2% isoflurane (Forane, AESICA, Queenborough Limited Kent, UK). Then, the chest was shaved, and the rat was placed in a supine position onto a heating pad. Two-dimensional, M-mode, Doppler, and tissue Doppler echocardiographic examinations were performed in accordance with the criteria of the American Society of Echocardiography with a Vivid 7 Dimension ultrasound system (General Electric Medical Systems) using a phased array 5.5–12 MHz transducer (10S probe) as described previously (16, 32, 33). Data of three consecutive heart cycles were analyzed (EchoPac Dimension software; General Electric Medical Systems) by an experienced investigator in a blinded manner. The mean values of three measurements were calculated and used for statistical evaluation.

### *Ex vivo* Cardiac Perfusions and Tissue Harvesting

The hearts were weighed after 5 min of *ex vivo* heart perfusion (34–36). Apical and basal parts of the heart were freshly frozen and used for biochemical measurements in every follow-up time point (Figure 1D). A cross-section of the whole heart at the ring of the papillae was cut and fixed in 4% buffered formalin for histological analysis at week 19 (Figure 1D). Following the removal of the heart, the presence of pleural fluid was checked and collected from the chest. Body and lung weights were also measured in every follow-up time point.

### Hematoxylin-Eosin Staining

Five-micrometer paraffin-embedded transverse cut sections of the formalin-fixed subvalvular area of the ventricles were stained with hematoxylin-eosin, in the samples collected at week 19. On these slides, myocardial fiber diameters were measured to verify the development of LVH as described previously (37, 38). Transverse transnuclear widths (cardiomyocyte diameter) were measured of 100 longitudinally oriented, mono-nucleated cardiomyocytes on left ventricle sections cut on the same plane (37, 38).

### Picrosirius Red Staining and Image Analysis

Five-micrometer paraffin-embedded transverse cut sections of the formalin-fixed subvalvular area of the ventricles were stained with picrosirius red staining in the samples collected at week 19, to assess cardiac fibrosis as described previously [Figure 1D; (16, 38)]. Histological slides were scanned with a Pannoramic P250 scanner (3D-Histech, Budapest, Hungary) and digital images at the magnification of ×40 and ×100 were captured. Medium-size vessels and their perivascular connective tissue sheet, the subepicardial and subendocardial areas were avoided as best as possible. The picrosirius red dyed images were analyzed with an in-house developed program as described previously (16, 38). This program determines the proportion of red pixels of heart sections using two simple color filters. For each Red-Green-Blue (RGB) pixel, the program calculates the color of the pixel in Hue-Saturation-Luminance (HSL) color space. The first filter is used for detecting red portions of the image. The second filter excludes any white (empty) or light gray (residual dirt on the slide) pixel from further processing using a simple RGB threshold. In this way, the program groups each pixel into one of two sets: pixels considered red and pixels considered green but neither red, nor white, nor gray. Red pixels in the first set correspond to connective tissue and fibrosis. Green pixels in the second set correspond to cardiac muscle. Dividing the number of elements in the first set by the number of elements in both sets gives the proportion of the connective tissue compartment of the heart area examined.

### MicroRNA Expression Profiling by qRT-PCR

Quantitative RT-PCR was performed with miR-specific primers to monitor miR expression as described earlier [Figure 1D; (22, 38)]. In the case of miR-212, RNA was isolated using Trizol reagent (Invitrogen, #15596-018) from heart tissue. For quantitative detection of miR-212, TaqMan MicroRNA Reverse Transcription Kit (Applied Biosystems, #4366597), TaqMan miR-212, and snoRNA (U64702) Assays (Applied Biosystems, #A25576 and #4427975), and Absolute Blue qPCR Mix (Abgene, #AB-4136/B) were used according to the manufacturer's instructions. SnoRNA was used as a control for normalization.

### mRNA Expression Profiling by qRT-PCR

Target mRNAs of miR-212 associated with cardiac hypertrophy and heart failure were selected in the TargetScan database. Quantitative RT-PCR was performed with gene-specific primers to monitor mRNA expression as described previously [Figure 1D; Table 1; (38, 39)]. RNA was isolated using Qiagen RNeasy Fibrous Tissue Mini Kit (Qiagen, #74704) from heart tissue. Briefly, 3 μg of total RNA was reverse transcribed using High-Capacity cDNA Reverse Transcription Kit (Applied Biosystems, #4368814), specific primers and FastStart Essential DNA Green Master (Roche, #06402712001) were used according to the manufacturer's instructions. Peptidyl prolyl isomerase A (Ppia), hypoxanthine phosphoribosyltransferase 1 (Hprt1), ribosomal protein lateral stalk subunit P2 (Rplp2), and glyceraldehyde-3-phosphate dehydrogenase (Gapdh) were used as controls for normalization (Table 1).

**Table 1:** Primer sequences.

**Gene name**	**Gene symbol**	**Forward primer sequence**	**Reverse primer sequence**
Peptidylprolyl isomerase A (cyclophilin A)	*Ppia*	tgctggaccaaacacaaatg	caccttcccaaagaccacat
Hypoxanthine phosphoribosyltransferase 1	*Hprt1*	gaccggttctgtcatgtcg	acctggttcatcatcactaatcac
Ribosomal protein lateral stalk subunit P2	*Rplp2*	agcgccaaagacatcaagaa	tcagctcactgatgaccttgtt
Glyceraldehyde-3-phosphate dehydrogenase (GAPDH)	*Gapdh*	gaagggctcatgaccacagt	ggatgcagggatgatgttct
Natriuretic peptide A (ANP)	*Nppa*	cacagatctgatggatttcaaga	cctcatcttctaccggcatc
Natriuretic peptide B (BNP)	*Nppb*	gtcagtcgcttgggctgt	ccagagctggggaaagaag
Myosin heavy polypeptide 6, cardiac muscle, alpha (α-MHC)	*Myh6*	cgaaactgaaaacggcaag	catagcgctccttgagattgt
Myosin heavy polypeptide 7, cardiac muscle, beta (β-MHC)	*Myh7*	atccctggatcaggacaaga	agcttcaggtcaccctcca
Protein phosphatase 3 catalytic subunit alpha	*Ppp3ca*	tgaggctgaaaaagcaatacg	aaaccctttgcctcttcaaaa
Protein phosphatase 3 catalytic subunit beta	*Ppp3cb*	tcagaagaagatggatttgacg	tgctcggatcttgttcctg
Nuclear factor of activated T-cells, cytoplasmic, calcineurin-dependent 4	*Nfatc4*	gggggctgtcaaggctgctc	gcgcccgatgtctgtctcacc
Muscle atrophy F-box protein (atrogin 1)	*Fbx32*	ccatcaggagaagtggatctatgtt	gttcatgaagttcttttgggcgatgc
Myocyte-enriched calcineurin-interacting protein 1 (MCIP1.4)	*Rcan1*	agctccctgattgcctgtgt	tttggccctggtctcacttt
Forkhead box O3	*Foxo3*	gctaagcaggcctcatctca	ttcggtcagtttgagggtct
Mitogen activated protein kinase 1 (ERK2)	*Mapk1*	tctgcaccgtgacctcaa	gcaaggccaaagtcacaga
Myocyte enhancer factor 2a	*Mef2a*	gcacacagagcaccttgtaga	ttaggagacaagtagtccaaggaag
Protein kinase AMP-activated catalytic subunit alpha 2 (AMPK)	*Prkaa2*	gacaatcggagctatcttctagactt	aggtgttgaagaaccagacctc
DnaJ heat shock protein family (Hsp40) member A2	*Dnaja2*	aggtgtgcgcattatgataaga	cggtctttttcattgatgacct
Sirtuin 1, transcript variant X1 (predicted)	*Sirt1*	ttcgtggagatatttttaatcaggt	ctggtaagttttcaccaaagaagac
Phosphatase and tensin homolog	*Pten*	catgagcgagttggtcaaga	ccatgctgtgctggttca
Collagen type III alpha 1	*Col3a1*	gaggaatgggtggctatcct	ggtatccaggagaaccaggag
Collagen type I alpha 1	*Col1a1*	gggtctagacatgttcagctttg	ggcagtggcccctaagag

### Matrix Metalloprotease 2 (MMP-2) Zymography

Cardiac MMP-2 activity was measured at week 19 from homogenized samples, to estimate the collagen breakdown in the cardiac extracellular matrix as we described previously (40, 41). Briefly, polyacrylamide gels were copolymerized with gelatin, and a 40 μg protein was separated by electrophoresis (150 V, 1.5 h) in each lane. Following electrophoresis, gels were washed with 2.5% Triton X-100 and incubated for 20 h at 37°C in incubation buffer. Gels were then stained with 0.05% Coomassie Brilliant Blue in a mixture of methanol/acetic acid/water and de-stained in aqueous 4% methanol/8% acetic acid. Zymograms were digitally scanned, and band intensities were quantified using Quantity One software (Bio-Rad, Hercules, CA) (41).

### Western Blot

To investigate gene expression changes at protein quantity and activity level, standard Western blot technique was used in case of phospho-AKT, AKT, phospho-FOXO3, and FOXO3 with GAPDH loading background at week 19 [Figure 1D; (38, 42, 43)]. Heart tissue samples (*n* = 7–8) were homogenized with an ultrasonicator (UP100H Hielscher, Teltow, Germany) in RIPA (Radioimmunoassay) buffer (50 mM Tris–HCl (pH 8.0), 150 mM NaCl, 0.5% sodium deoxycholate, 5 mM EDTA, 0.1% SDS, 1% NP-40 (Cell Signaling, Carlsbad, CA, USA) supplemented with protease inhibitor cocktail and phosphatase inhibitors PMSF and NaF (Sigma, Saint Louis, MO, USA). The crude homogenates were centrifuged at 15,000 × g for 30 min at4°C. After quantification of protein concentrations of the supernatants, using the BCA Protein Assay Kit (Pierce, Rockford, IL, USA), 25 μg reduced and denaturated protein was loaded and SDS-PAGE (10% gel, 90 V, 2 h) was performed followed by the transfer of proteins onto a nitrocellulose membrane (20% methanol, 35 V, 2 h). The efficacy of the transfer was checked using Ponceau staining. The membranes were cut horizontally into three parts corresponding to the molecular weights of AKT, FOXO3, and GAPDH. Then the membranes were blocked for 1 h in 5% (w/v) BSA at room temperature and then incubated with primary antibodies (Cell Signaling, Beverly, MA, USA; overnight, 4°C, 5% BSA) in the concentrations of 1:1,000 against AKT (#9272), phosho-AKT (Ser473, #4060), phospho-FOXO3 (Ser253; #13129), 1:500 against FOXO3 (#2497) or 1:5,000 against GAPDH (#2118 overnight, 4°C, 1% BSA). Then the membranes were incubated with horseradish peroxidase (HRP)-conjugated goat anti-rabbit secondary antibody 1:2,000 (1:1,000 for FOXO3, 1:5,000 for GAPDH) (Dako Corporation, Santa Barbara, CA, USA; 45 min, room temperature, 1% BSA). After assessment of phosphorylated proteins, the membranes were stripped and reassessed for the total amount of proteins. An enhanced chemiluminescence kit (Cell Signaling, Carlsbad, CA, USA) was used to develop the membranes. The chemiluminescence signals were analyzed and evaluated by Quantity One Software. Signals of GAPDH and FOXO3, as well as AKT, develop at different time points. Therefore, the expositions times are different for GAPDH, FOXO3, and AKT. For signal evaluation of Western blots, we always used the non-oversaturated films for correct signal detection (Supplementary Figures).

### Statistical Analysis

Statistical analysis was performed using Sigmaplot 12.0 for Windows (Systat Software Inc.). All values are presented as mean ± SEM except the gene expression data measured by qRT-PCR. Data showed normal distribution unless otherwise indicated. Data measured at different follow-up time points in separate experiments including body weight, heart weight, heart weight to body weight ratio, lung weight and echocardiographic parameters were compared using One-Way ANOVA between the groups. A Bonferroni test was used as a *post-hoc* test. A two sample *t*-test (in case of the normal distribution of the data) or Mann Whitney *U*-test (in case of the non-normal distribution of the data) was used to determine the effect of RIHD on all measured parameters within each time point. *P* < 0.05 was accepted as a statistically significant difference. In the case of target genes, the analysis of relative gene expression data was performed using the 2^−Δ*ΔCt*^ method. Gene expression ratios with a *p* < 0.05 and fold change of <−2.00 or fold change of >2.00 were considered as repression or overexpression respectively in gene activity.

## Results

### Characteristics of the RIHD Models

At week 1, there was no difference in the body weight between the control and irradiated groups (Table 2). At week 3, the irradiated animals presented a trend toward a lower body weight as compared to the time-matched controls (Table 2). At week 19, the irradiated rats showed significantly lower body weight as compared to the time-matched controls (Table 2). At autopsy at week 1 and 3, there was no pleural fluid in the irradiated animals. At week 19, the presence of extensive pleural fluids was found in almost all animals in the irradiated group (data not shown). At the macroscopic level, no major heart pathologies (including the large vessels, the valves, the coronary arteries, etc.) were observed in the irradiated groups at any follow-up time point. At week 1 and 3, lung weights were significantly increased in the irradiated groups as compared to the time-matched controls (Table 2). A higher lung weight might suggest the presence of acute inflammation and lung edema due to the acute deteriorating effects of ionizing radiation. At week 19, no difference was found in lung weights between the groups (Table 2). At week 1 and 3, there was no difference in heart weights between the irradiated groups and their time-matched controls (Table 2). However, the heart to body weight ratio showed a trend toward an increase in the irradiated group at week 3 (Table 2). The increased heart to body weight ratio might suggest the development of starting cardiac hypertrophy. At week 19, the tibia length (4.23 ± 0.05 vs. 4.46 ± 0.04 cm, *p* = 0.003) and the heart weight was significantly lower in the irradiated group than in the control group pointing out the growth retardation in these animals (Table 2). At week 19, no sign of radiation-induced pneumonitis or lung fibrosis was visible, confirming the appropriateness of our models for the study of the acute and chronic effects of selective heart irradiation. At week 19, all of the morphometric parameters (body weight, heart weight, heart weight to body weight ratio, and lung weight) were significantly higher in both the control and irradiated groups as compared to week 1 parameters within the same group, due to the growth of the animals (Table 2).

**Table 2:** Characteristics of the RIHD models at week 1, 3, and 19, respectively.

**Parameter (unit)**	**Week 1**			**Week 3**			**Week 19**		
	**Control**	**RT**	***p*-value**	**Control**	**RT**	***p*-value**	**Control**	**RT**	***p*-value**
Body weight at the endpoint (g)	270 ± 9	254 ± 8	0.205	394 ± 11^#^	366 ± 10^#^	0.091	597 ± 24^#^	425 ± 43^#^^*^	0.004
Heart weight (g)	1.21 ± 0.07	1.13 ± 0.05	0.340	1.33 ± 0.05	1.45 ± 0.09^#^	0.286	1.74 ± 0.08^#^	1.34 ± 0.06^#^^*^	0.002
Lung weight (g)	1.35 ± 0.06	1.64 ± 0.07^*^	0.006	2.01 ± 0.09^#^	2.72 ± 0.25^#^^*^	0.014	2.29 ± 0.17^#^	2.26 ± 0.18^#^	0.883
Heart weight/body weight ratio^*^1,000	4.45 ± 0.13	4.44 ± 0.10	0.918	3.38 ± 0.10^#^	3.99 ± 0.32^#^	0.100	2.92 ± 0.09^#^	3.36 ± 0.35^#^	0.239

*Values are mean ± SEM, n = 8*,

* *p < 0.05 vs. control, unpaired t-test within the same time points. p-values refer to the unpaired t-test at each time point*.

# *p < 0.05 vs. week 1 within the same group (control or RT), One-Way ANOVA, Bonferroni post-hoc test. RT, radiotherapy*.

### HFpEF Developed in RIHD at Week 19

**Figure 2:**
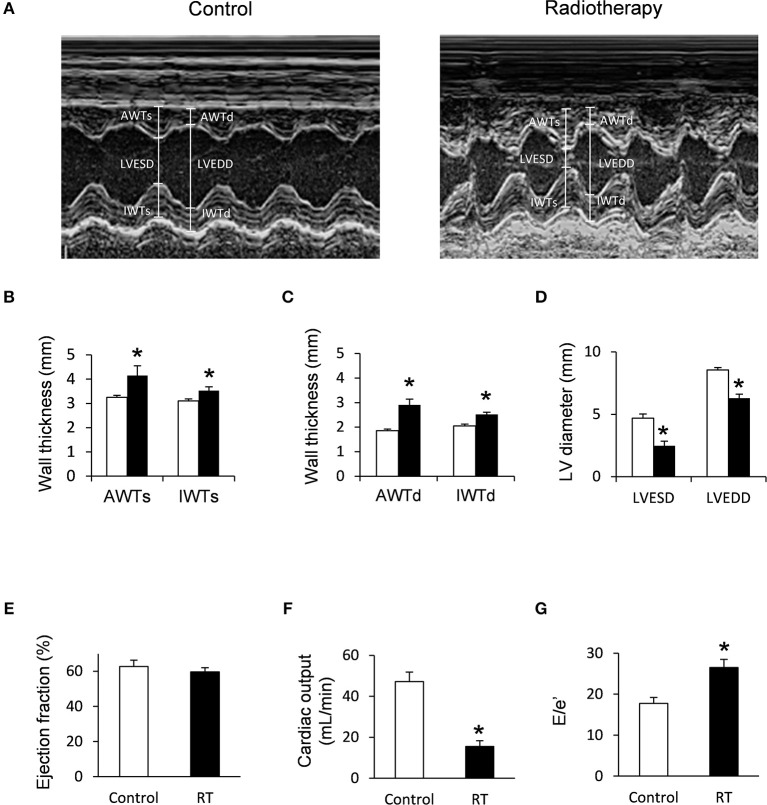
Echocardiographic results at week 19. **(A)** Representative M-mode images, **(B)** anterior and inferior wall thicknesses in systole (AWTs and IWTs), **(C)** anterior and inferior wall thicknesses in diastole (AWTd and IWTd), **(D)** left ventricular end systolic diameter (LVESD) and left ventricular end diastolic diameter (LVEDD), **(E)** ejection fraction, **(F)** cardiac output, and **(G)** E/e' ratio. White bars represent control group and black bars represent the irradiated group. RT means radiotherapy. Values are means ± SEM, *n* = 8, *p < 0.05.

Transthoracic echocardiography was performed at week 1, 3, and 19 in separate experiments to investigate whether the development of acute and chronic RIHD leads to an alteration in myocardial morphology and function (Table 3; Figures 1A, 2).

**Table 3:** Effects of radiotherapy on various *in vivo* left ventricular morphological and functional parameters measured by transthoracic echocardiography at week 1, 3, and 19, respectively.

**Parameter (unit)**	**View/Mode**	**Week 1**			**Week 3**			**Week 19**		
		**Control**	**RT**	***p*-value**	**Control**	**RT**	***p*-value**	**Control**	**RT**	***p*-value**
Posterior wall thickness-systolic (mm)	Long axis/MM	2.78 ± 0.12	2.95 ± 0.11	0.307	2.94 ± 0.11	3.16 ± 0.12	0.214	3.19 ± 0.16^#^	3.37 ± 0.24^#^	0.526
Posterior wall thickness-diastolic (mm)	Long axis/MM	1.85 ± 0.07	1.76 ± 0.07	0.357	2.01 ± 0.12	1.80 ± 0.13	0.122	2.00 ± 0.11	2.44 ± 0.14^#^^*^	0.026
Septal wall thickness-systolic (mm)	Long axis/MM	2.91 ± 0.13	3.06 ± 0.08	0.349	3.13 ± 0.08	3.54 ± 0.13^*^	0.017	3.27 ± 0.13^#^	3.71 ± 0.29^#^	0.186
Septal wall thickness-diastolic (mm)	Long axis/MM	1.64 ± 0.07	1.79 ± 0.10	0.233	1.65 ± 0.05	1.85 ± 0.04^*^	0.009	1.87 ± 0.12^#^	2.69 ± 0.34^#^^*^	0.037
Left ventricular end diastolic diameter (mm)	Long axis/MM	7.73 ± 0.10	7.31 ± 0.15^*^	0.035	7.99 ± 0.13	7.63 ± 0.25	0.207	8.55 ± 0.14^#^	6.04 ± 0.52^#^^*^	0.000
Left ventricular end systolic diameter (mm)	Long axis/MM	4.45 ± 0.20	3.76 ± 0.23^*^	0.048	4.37 ± 0.16	3.50 ± 0.26^*^	0.011	4.13 ± 0.23	2.59 ± 0.55^#^^*^	0.021
Fractional shortening (%)	Long axis/MM	43 ± 3	48 ± 3	0.152	45 ± 2	54 ± 3^*^	0.020	46 ± 1	63 ± 6^#^^*^	0.013
Left ventricular end-diastolic volume (μl)	Four chamber/2D	135 ± 15	94 ± 6^*^	0.023	120 ± 9	114 ± 8	0.646	262 ± 27^#^	91 ± 13^*^	0.000
Left ventricular end-systolic volume (μl)	Four chamber/2D	54 ± 6	33 ± 3^*^	0.008	38 ± 3^#^	37 ± 3	0.871	100 ± 17^#^	36.±5.^*^	0.003
Stroke volume (μl)	Four chamber/2D	82 ± 9	61 ± 5	0.064	82 ± 6	77 ± 6	0.676	162 ± 14^#^	56 ± 9^*^	0.000
Heart rate (beats/min)	Four chamber/2D	349 ± 10	373 ± 6	0.052	338 ± 14	352 ± 14	0.488	291 ± 7^#^	279 ± 7^#^	0.278
E/e'-wave (m/s)	4 chamber/PWD	13 ± 1	23 ± 1^*^	0.002	15 ± 1	30 ± 3^*^	0.000	17 ± 1	25 ± 2	0.004
e'-wave (m/s)	4 chamber/TD	0.079 ± 0.005	0.042 ± 0.002^*^	0.009	0.071 ± 0.007	0.038 ± 0.003^*^	0.006	0.055 ± 0.004^#^	0.042 ± 0.005^*^	0.009
Maximal LV outflow tract velocity (m/s)	4 chamber/PWD	3.81 ± 0.38	4.38 ± 0.71	0.496	2.57 ± 0.36	2.10 ± 0.33^#^	0.363	4.11 ± 0.63	2.04 ± 0.54^#^^*^	0.030
Mean LV outflow tract velocity (m/s)	4 chamber/PWD	2.60 ± 0.30	2.98 ± 0.49	0.518	1.62 ± 0.23	1.53 ± 0.26^#^	0.548	2.50 ± 0.41	1.28 ± 0.36^#^^*^	0.046

*Transthoracic echocardiographic measurements were performed 1, 3, and 19 weeks after the selective heart irradiation in separated experiments. Values are mean ± SEM, n = 8*,

* *p < 0.05 vs. control, unpaired t-test within the same time point. p-values refer to the unpaired t-test at each time point*.

# *p < 0.05 vs. week 1 within the same group (control or RT), One-Way ANOVA, Bonferroni post-hoc test. 2D, two-dimensional, E-wave, early ventricular filling velocity; LV, left ventricular, MM, M (motion) Mode; PW, pulse wave; RT, radiotherapy; TD, tissue Doppler*.

At week 1, the left ventricular wall thicknesses were not different between the irradiated and time-matched control groups (Table 3). In contrast, the left ventricular end-systolic and end-diastolic diameters, as well as the volumes, were significantly decreased in the irradiated animals, proving an acute dysfunction in the contraction and relaxation as well (Table 3). Stroke volume showed a statistically non-significant trend toward a decrease in the irradiated animals as compared to the time-matched controls (Table 3). There was a trend toward an increase in heart rate in the irradiated animals, likely a compensatory mechanism, to maintain cardiac output (Table 3). The ratio of the early flow velocity E and the septal mitral annulus velocity e′ significantly increased, and e′ significantly decreased in irradiated rats indicating the presence of concomitant diastolic dysfunction (Table 3).

At week 3, septal wall-thicknesses both in diastole and systole were significantly increased in the irradiated rats indicating the development of LVH (Table 3). The left ventricular end-systolic diameter was significantly decreased, and fractional shortening was significantly increased in the irradiated animals in this early phase of hypertrophy (Table 3). Diastolic dysfunction was also present in the irradiated group characterized by significantly increased E/e′ and significantly decreased e′ (Table 3).

At week 19, left ventricular wall thicknesses including anterior and inferior walls both in systole and diastole, as well as septal and posterior walls in diastole, significantly increased in irradiated rats pointing to the presence of a marked concentric LVH (Table 3; Figures 2A–C). In addition, both left ventricular end-systolic and end-diastolic diameters significantly decreased in irradiated animals as compared to the controls (Figure 2D). Indeed, left ventricular end-systolic, as well as end-diastolic volumes also decreased in irradiated rats (Table 3). In contrast, ejection fraction remained unchanged in irradiated rats showing a characteristic picture of HFpEF (Figure 2E). There was no difference in heart rate between the two groups at this stage (Table 3). Stroke volume and cardiac output were significantly reduced in irradiated rats as compared to controls (Table 3; Figure 2F). More importantly, e′ was significantly decreased and E/e′ was significantly increased indicating diastolic dysfunction (Table 3; Figure 2G).

Septal and posterior wall thicknesses were significantly increased both in the control and irradiated group at week 19 as compared to data measured at week 1 in the same group, due to the growth of the animals (Table 3). Interestingly, left ventricular end-diastolic and end-systolic diameters were significantly decreased in the RIHD group at week 19 as compared to week 1 values, indicating the presence of marked concentric hypertrophy (Table 3). In the control group, left ventricular end-diastolic diameter was significantly increased at week 19 as compared to week 1 values, due to the normal growth of the animals (Table 3). Heart rate was significantly decreased in both the control and irradiated groups at week 19 as compared to week 1 values which might be caused by the aging of the animals (Table 3).

### Cardiomyocyte Hypertrophy and Interstitial Fibrosis in RIHD at Week 19

**Figure 3:**
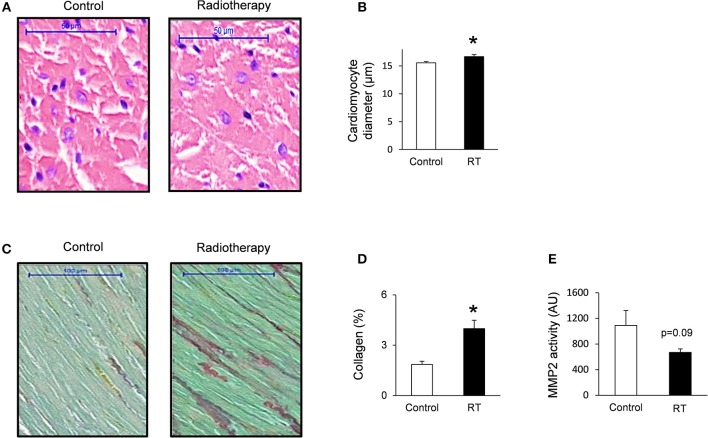
Histology and zymography results at week 19. **(A)** Hematoxylin-eosin stained slides, **(B)** cardiomyocyte diameter, **(C)** picrosirius red and fast green stained slides, **(D)** cardiac collagen content, and **(E)** MMP2 activity. White bars represent control group and black bars represent the irradiated group. RT means radiotherapy. Values are means ± SEM, *n* = 8, *p < 0.05.

To verify the development of severe LVH seen on echocardiographic images at week 19, cardiomyocyte diameters were measured histologically (Figures 3A,B). Cross-sectional cardiomyocyte diameters significantly increased in RIHD proving the presence of LVH at the cellular level at week 19 (Figure 3B).

Collagen deposition was assessed to investigate the development of fibrosis in response to selective heart irradiation at week 19 (Figures 3C,D). Significant interstitial fibrosis was found with slight but consistent collagen depositions in all studied segments of the irradiated hearts (Figures 3C,D). MMP2 activity also showed a decreasing trend at week 19 (Figure 3E), suggesting that the balance between collagen breakdown and deposition might be shifted toward deposition.

### Molecular Markers of Cardiac Hypertrophy and Fibrosis

The expression of cardiac hypertrophy (alpha-MHC and beta-MHC), fibrosis (collagen type I alpha 1 - *Col1a1*- and collagen type III alpha 1- *Col3a1*-) and heart failure (A-type natriuretic peptide [ANP] and B-type natriuretic peptide [BNP]) markers were measured by qRT-PCR at week 1, 3, and 19 (Tables 4, 5). At week 1 and 3, the cardiac expression of these markers showed no difference among the irradiated and time-matched control groups (Table 4).

**Table 4:** Cardiac gene expression changes 1 and 3 weeks after the selective heart irradiation (qRT-PCR results).

**Gene name**	**Gene symbol**	**Week 1**				**Week 3**			
		**Log_**2**_ change**	**SD log_**2**_ change**	***p*-value**	**Fold change**	**Log_**2**_ change**	**SD log_**2**_ change**	***p*-value**	**Fold change**
miR-212	*miR-212*	−0.38	0.63	0.183	−1.30	1.11	0.38	0.000	2.15^*^
Forkhead box O3	*Foxo3*	−0.02	0.41	0.916	−1.01	−0.19	0.29	0.170	−1.14
Myosin, Heavy Polypeptide 6, Cardiac Muscle, Alpha (α-MHC)	*Myh6*	−0.45	0.29	0.009	−1.37	−0.38	0.61	0.193	−1.30
Myosin, Heavy Polypeptide 7, Cardiac Muscle, Beta (β-MHC)	*Myh7*	−0.13	0.23	0.237	−1.09	−0.12	0.59	0.666	−1.08
Collagen, type III, alpha 1	*Col3a1*	−0.52	0.61	0,079	−1.44	1.11	0.97	0.027	1.24
Collagen, type I, alpha 1	*Col1a1*	−0.43	0.46	0.171	−1.37	0.39	0.59	0.171	1.31
Natriuretic peptide A (ANP)	*Nppa*	0.76	1.26	0.198	1.71	0.56	0.97	0.231	1.47
Natriuretic peptide B (BNP)	*Nppb*	−0.09	0.75	0.789	−1.08	0.55	0.76	0.132	1.46

Gene expression ratios at week 1 and 3, respectively, (radiotherapy vs. control). Fold change of < −2.00 or >2.00 (repression or overexpression respectively) and a p < 0.05 were considered as a significant change

* *. n = 6, unpaired t-test or Mann-Whitney U-test*.

**Table 5:** Cardiac gene expression changes 19 weeks after the selective heart irradiation (qRT-PCR results).

	**Gene name**	**Gene symbol**	**Log_**2**_ change**	**SD log_**2**_ change**	***p*-value**	**Fold change**
miR-212 targets	Forkhead box O3	*Foxo3*	−1.01	1.11	0.000	−2.01^*^
	Mitogen activated protein kinase 1 (ERK2)	*Mapk1*	−0.47	0.34	0.000	−1.39
	Myocyte enhancer factor 2a	*Mef2a*	−0.10	0.52	0.051	1.10
	Protein kinase AMP-activated catalytic subunit alpha 2 (AMPK)	*Prkaa2*	−0.49	0.46	0.000	−1.41
	DnaJ heat shock protein family (Hsp40) member A2	*Dnaja2*	−0.41	0.43	0.000	−1.33
	Sirtuin 1. transcript variant X1	*Sirt1*	−0.34	0.38	0.000	−1.26
	Protein phosphatase 3 catalytic subunit alpha (Calcineurin A-alpha)	*Ppp3ca*	−0.45	0.61	0.000	−1.37
	Phosphatase and tensin homolog (PTEN)	*Pten*	−0.04	0.27	0.408	−1.03
Other Genes	Myosin heavy polypeptide 6, cardiac muscle, alpha (α-MHC)	*Myh6*	−2.04	1.08	0.007	−4.11^*^
	Myosin heavy polypeptide 7 cardiac muscle, beta (β-MHC)	*Myh7*	−0.62	0.33	0.003	−1.53
	Collagen type III alpha 1	*Col3a1*	−1.45	0.87	0.000	−2.73^*^
	Collagen type I alpha 1	*Col1a1*	0.14	0.72	0.253	1.10
	Protein phosphatase 3 catalytic subunit beta (Calcineurin A-beta)	*Ppp3cb*	−0.44	0.24	0.000	−1.36
	Muscle atrophy F-box protein (Atrogin-1)	*Fbxo32*	−0.17	0.25	0.159	−1.12
	Myocyte-enriched calcineurin-interacting protein 1 (MCIP1.4)	*Rcan1*	−0.18	0.51	0.455	−1.13
	Natriuretic peptide A (ANP)	*Nppa*	3.88	1.80	0.000	14.77^*^
	Natriuretic peptide B (BNP)	*Nppb*	1.50	0.53	0.000	2.83^*^

Gene expression ratios at week 19 (radiotherapy vs. control). Fold change of < −2.00 or >2.00 (repression or overexpression. respectively) and a p < 0.05 were considered as a significant change

* *. n = 6, unpaired t-test or Mann-Whitney U-test*.

At week 19, the beta-MHC to alpha-MHC ratio showed a 2.7-fold increase among cardiac hypertrophy markers. The cardiac expression of alpha-MHC significantly decreased in RIHD as compared to the controls (Table 5). However, the cardiac beta-MHC mRNA level did not change in RIHD as compared to controls. The cardiac expression of pro-hypertrophic protein phosphatase 3, catalytic subunit alpha and beta (i.e., calcineurin A-alpha and calcineurin A-beta), myocyte enhancer factor 2D (*Mef2d*), and myocyte enhancer factor 2C (*Mef2c*) did not change in response to selective heart irradiation as compared to the controls (Table 5). The anti-hypertrophic atrogin-1 and the pro-hypertrophic calcineurin pathway element myocyte-enriched calcineurin-interacting protein 1.4 (MCIP 1.4) did not change significantly in RIHD as compared to the controls (Table 5). Among fibrosis and remodeling markers, the more elastic *Col3a1* showed significant down-regulation in RIHD; however, the more rigid *Col1a1* failed to change in RIHD (Table 5). Consistently, the ratio of *Col1a1* to *Col3a1* increased, which is a characteristic change in left ventricular fibrosis and remodeling (44–46). Moreover, the mRNA levels of the heart failure markers, ANP and BNP, significantly increased in RIHD as compared to the controls (Table 5).

### Cardiac Overexpression of miR-212 at Week 3 and 19

**Figure 4:**
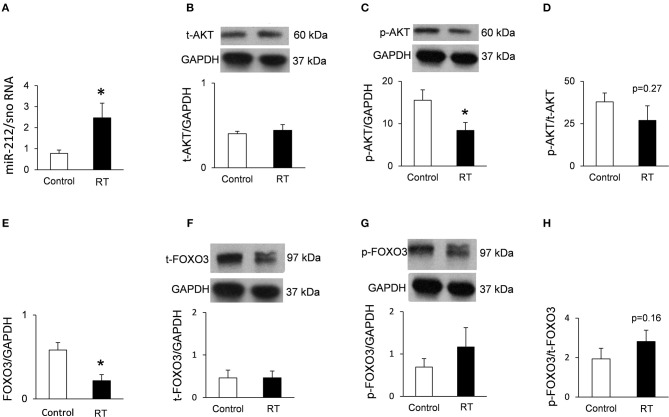
qRT-PCR and Western blot results at week 19. **(A)** Cardiac miR-212 expression, **(B)** cardiac total AKT expression, **(C)** cardiac phospho-AKT expression, **(D)** cardiac phospho-AKT/total AKT ratio, **(E)** cardiac FOXO3 mRNA expression, **(F)** cardiac total FOXO3 expression, **(G)** cardiac phospho-FOXO3 expression, and **(H)** cardiac phospho-FOXO3/total FOXO3 ratio. White bars represent control group and black bars represent the irradiated group. RT means radiotherapy. Values are means ± SEM, *n* = 7–8, *p < 0.05. In case of Western blot results, only the representative blot image was shown, and the mean was calculated.

At week 1, there was no change in the cardiac expression of miR-212 between the irradiated and control groups (Table 5). Both at week 3 and 19, miR-212 was significantly overexpressed in the irradiated hearts as compared to the controls (Table 4; Figure 4A).

### Repression of the Anti-hypertrophic FOXO3 at the mRNA Level in RIHD Only at Week 19

Both at week 1 and 3, there was no change in the cardiac expression of the anti-hypertrophic miR-212 target molecule FOXO3 between the irradiated and time-matched control groups (Table 4). In contrast, cardiac expression of FOXO3 was significantly decreased at the mRNA level in irradiated hearts as compared to controls at week 19 (Table 5; Figure 4E).

### Phospho-FOXO3/Total FOXO3 Ratio in RIHD at Week 19

**Figure 5:**
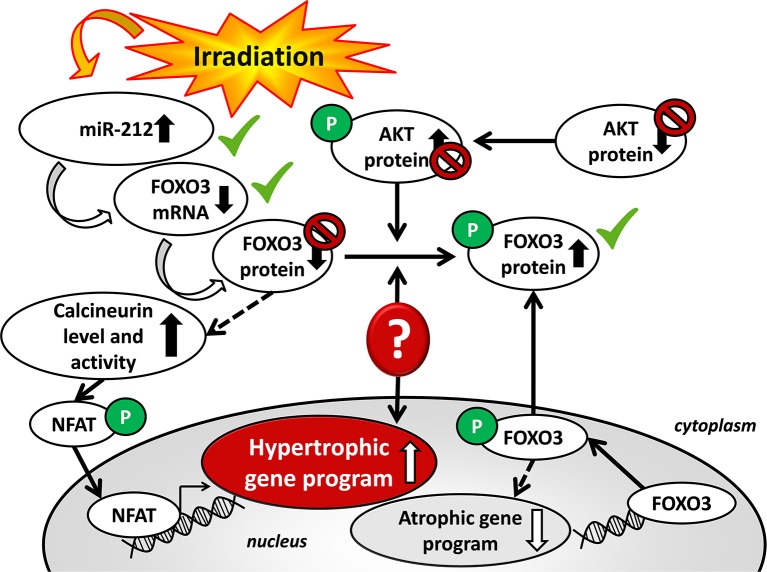
Putative mechanisms in the development of left ventricular hypertrophy in RIHD.

The expression of the anti-hypertrophic miR-212 target molecule FOXO3 was also investigated at the protein level using a Western blot technique at week 19. Total FOXO3 protein levels failed to decrease in the irradiated hearts as compared to the controls (Figure 4F). The cardiac phospho-FOXO3 level and the phospho-FOXO3/total FOXO3 ratio showed a statistically non-significant trend toward an increase in RIHD as compared to the control group (Figures 4G,H). An increased phospho-FOXO3/total FOXO3 ratio is considered to be a characteristic shift in pressure-overload-induced cardiac hypertrophy forms (47). In our RIHD model, the statistically non-significant trend toward an increase in the phospho-FOXO3/total FOXO3 ratio seems to be independent of the effect of miR-212 in RIHD (Figure 5).

### Decreased Cardiac phospho-AKT Level in RIHD at Week 19

Moreover, we investigated the cardiac amount of both total AKT and phospho-AKT proteins using Western blot. Phospho-AKT is a positive regulator of the FOXO3 protein phosphorylation, leading to a pro-hypertrophic shift in the phospho-FOXO3/FOXO3 ratio in pressure-overload-induced LVH models [Figure 5; (48)]. In our RIHD model, the cardiac amount of total AKT did not differ between the two groups (Figure 4B). However, the amount of phospho-AKT was significantly decreased in the irradiated hearts as compared to the controls (Figure 4C). The phospho-AKT/total AKT ratio showed a statistically non-significant trend toward a decrease in cardiac RIHD samples (Figure 4D). Hence, AKT does not seem to play a crucial role in the regulation of FOXO3 in RIHD (Figure 5).

### Hypertrophy Associated mRNA Targets Beyond FOXO3 in RIHD at Week 19

Several predictive miR-212 targets associated with hypertrophy have been identified beyond FOXO3. Among those, we have also investigated the cardiac expression of the extracellular signal-regulated kinase 2 (ERK2), the myocyte enhancer factor 2a (*Mef2a*); the protein kinase AMP-activated catalytic subunit alpha 2 (AMPK); DnaJ heat shock protein family member A2 (*Hsp40*); sirtuin 1, transcript variant X1 (*Sirt1*); protein phosphatase 3 catalytic subunit alpha (calcineurin A-alpha); phosphatase and tensin homolog (PTEN) (Table 5). These target mRNAs failed to show significant down-regulation in response to selective heart irradiation as compared to the control hearts (Table 5).

## Discussion

We have previously described the rat model of RIHD in the chronic phase (16). In our present study, we have characterized the rat model of RIHD in the acute phase, 1 and 3 weeks after selective heart irradiation. We also confirm the presence of diastolic dysfunction in this model in every investigated phase (49). In our present study, mild septal hypertrophy was found 3 weeks after the selective heart irradiation. Marked cardiac morphological and functional changes were confirmed 19 weeks after the selective heart irradiation, in the form of concentric LVH and diastolic dysfunction, which are the hallmarks of HFpEF. These morphological and functional changes were similar to those reported in other studies (1, 7).

Two major components are thought to be responsible for the development of RIHD in humans. One is the progressive atherosclerosis of the coronary arteries due to endothelial damage (5, 6). The other is diffuse injury and inflammation of the myocardium caused by the loss of capillaries and cardiomyocytes (9). These mechanisms could lead to compensatory cardiomyocyte hypertrophy and interstitial fibrosis in the early stages of RIHD (5, 6). The injury of coronary arteries is difficult to test in wild-type rodent models due to different serum lipid profiles (i.e., higher HDL-cholesterol and lower LDL-cholesterol levels as compared to humans) which naturally protect them from the development of atherosclerosis (50). In our present study, the lack of atherosclerosis makes it possible to study the direct effects and mechanisms of diffuse myocardial injury and chronic inflammation components. Available studies are mainly focused on macro- and microvascular contributions in RIHD (3, 6, 10, 11). So far only a few studies investigated the molecular mechanisms of radiation-induced diffuse myocardial injury and compensatory hypertrophy (1, 7, 15).

In our present study, picrosirius red staining indicates an increased interstitial accumulation of collagens in the RIHD hearts. Generally, the increased collagen deposition could be explained by either the stimulation of production or the reduced turnover of the mRNAs and/or proteins of different collagen types. In our RIHD model, we could not demonstrate increased expressions of collagens at the mRNA level at week 19, only a decrease in *Col3a1* and no change in *Col1a1* expression. Likely, the excessive stimulation of collagen expression happens at the mRNA level at earlier time points, and only the result of an earlier stimulation was visible at the protein level in our study. However, we observed a trend toward reduced MMP-2 activity in RIHD hearts at week 19, which might indicate a reduced turnover of collagens at the protein level. There was no change in the cardiac gene expression of *Col1a1* and *Col3a1* in the early phases of RIHD. Therefore, the 1- and 3-week follow-up times seem to be too early to detect significant stimulation of collagen gene expression at the mRNA level in RIHD. The key mediator of radiation-induced cardiac fibrosis is thought to be a transforming growth factor beta (TGF-β1) (15, 51). In our previous experiments, increased plasma levels of TGF-β1 and growth differentiation factor 15 (GDF-15) were shown 12 weeks after the selective heart irradiation with 50 Gy (16). Another possible explanation is that our RIHD model in week 19 is not in the phase of severe fibrotic remodeling, which ultimately leads to systolic heart failure with reduced ejection fraction (HFrEF). According to our experimental data, the heart at 19 weeks following irradiation is best characterized by hypertrophy, mild interstitial fibrosis, and diastolic dysfunction, demonstrating characteristic HFpEF. Another study demonstrated that the degeneration of cardiomyocytes extended until 100 days after the irradiation (15). In our chronic RIHD model, the mild interstitial fibrosis might be developed as a reaction to diffuse cardiomyocyte cell death (52). This replacement fibrosis is thought to be essential in the adaptation to the loss of cardiac parenchyma and the preservation of the heart structure in different heart failure models (52). Thus, more severe fibrosis might be detectable at a later stage (>19 weeks), followed by dilatation of the heart, thinner walls and end-stage heart failure with reduced ejection fraction in RIHD (15). A study with a 16-month follow-up time reported that interstitial collagen deposition had a progressive nature (15).

It is also interesting to note that the gene expression of type III collagen fibers was significantly repressed, and the expression of type I collagen fibers were not changed in response to selective heart irradiation in our present study at week 19. Collagen type III is of the thinner and more elastic fiber type, whereas collagen type I is of the thicker and more rigid fiber type (44). The increased ratio of *Col1a1* to *Col3a1* at the protein level could be a factor in the stiffness and resistance to stretch in both in HFpEF and HFrEF in different heart failure and hypertrophy forms (44–46).

Unfortunately, therapeutic options for RIHD are currently insufficient. Since interstitial fibrosis is a final major endpoint of RIHD, our chronic model seems to be an adequate one, in a series of future investigations with different anti-fibrotic agents to prevent fibrosis in an early phase. Interestingly, a recent study showed cardioprotective effects of an endogenously released small peptide N-acetyl-Ser-Asp-Lys-Pro (Ac-SDKP) in a rat model of RIHD. In this experimental study, Ac-SDKP was reported to inhibit inflammation, fibrosis, and reduce macrophage activation (53).

A number of studies demonstrated that miRs are critical contributors to cardiovascular biology and disease development (18–21, 54). So far, only two studies have been published, describing that miR-1, as well as miR-15b, were repressed and miR-21 was overexpressed in RIHD (55, 56). It has been demonstrated that hypertrophic stimuli upregulated cardiac expression of miR-212, which is necessary and sufficient to drive the hypertrophic growth of cardiomyocytes (22). MiR-212 was shown to be a key regulator of the development of LVH and heart failure via the repression of the anti-hypertrophic transcription factor FOXO3 and overactivation of the calcineurin/NFAT signaling during heart failure development (22). In our present study, LVH was accompanied by the overexpression of miR-212 3 and 19 weeks after the selective heart irradiation. Interestingly, the repression of FOXO3 at the mRNA level was present in cardiac tissue samples in RIHD only at week 19 (Figure 5). In contrast, the FOXO3 protein level failed to decrease, and the phospho-FOXO3/total FOXO3 ratio showed a non-significant trend toward an increase in RIHD at week 19 (Figure 5). Although, the increased phospho-FOXO3/total FOXO3 ratio is a characteristic shift in cardiac hypertrophy forms (47); it seems to be independent of the effect of miR-212 in chronic RIHD. FOXO transcription factors are also regulated by the protein kinase AKT [Figure 5; (48, 57)]. Therefore, we also measured the protein levels of both total and phospho-AKT at week 19. The total-AKT level and the phospho-AKT/total AKT ratio failed to change significantly in the irradiated hearts as compared to the controls. It has also been demonstrated in other LVH models that the repression of the FOXO3 level could indirectly lead to the overactivity of the hypertrophic calcineurin/NFAT pathway (57). In our present study, cardiac mRNA levels of calcineurin A-alpha and calcineurin A-beta showed a statistically non-significant decrease in the irradiated hearts as compared to the controls. These results suggest that the AKT/FOXO3 mediated pathways might not play a crucial role in the development of LVH in chronic RIHD. Nonetheless, the FOXO3 protein level and its phosphorylation are also regulated by various molecules beyond miR-212.

In our chronic RIHD model, we also investigated the expression of selected regulatory genes that are predicted targets of miR-212 and are associated with cardiac hypertrophy in other LVH forms. Target mRNAs were Mef2a, AMPK, Hsp40, sirtuin 1, calcineurin A-alpha, PTEN, and ERK2. These target mRNAs failed to show a significant change in RIHD as compared to the control hearts. Therefore, we did not further investigate their cardiac expression at the protein level.

The development of the LVH and HFpEF in RIHD seems to be unique and very different from other forms of cardiac hypertrophies, such as the commonly studied pressure-overload-induced compensatory hypertrophy and subsequent heart failure. In RIHD, compensatory hypertrophy, cell survival, and recovery mechanisms could be activated simultaneously in a distinct manner. These individual pathways may converge in the same direction toward key down-stream regulators (e.g., FOXO3) and the ensuing opposite effects may extinct each other at the protein level.

In this study, the focus was on the miR-212 with its selected hypertrophy-associated target mRNA molecules, the relative role of other miRNAs or other miR-212 targets were not assessed in the development of cardiac hypertrophy in RIHD.

In summary, this is the first study to report that LVH and HFpEF are accompanied by characteristic overexpression of miR-212 in the heart, both in the early and later phases of RIHD. Our results suggest that miR-212 might play a significant role in the development of RIHD via FOXO3/AKT independent mechanisms, and the molecular mechanism of the development of RIHD is distinct from other forms of hypertrophy and pathological remodeling.

## Limitations

Our results regarding altered cardiac gene expression due to RIHD are based on selected miRNAs and mRNA target molecules, however, measurement of the full rat transcriptome should be performed in the future. Our study is descriptive; therefore, future studies providing more in-depth mechanistic insights should be carried out. Moreover, therapeutic intervention was out of the scope of our present exploratory study.

## Author Contributions

MS coordinated the study, performed transthoracic echocardiography and qRT-PCR for miR-212, evaluated experimental data, drafted, proofread, and edited the manuscript. RG and PD performed Western blot experiments. ÁZ performed qRT-PCR for mRNA targets. MS, LK, and GF treated the animals, isolated organs, and prepared samples. ZV and ZK planned and performed irradiation. BK and GC performed HE and picrosirius red staining and analyzed images. MK measured cardiomyocyte diameters. GS performed MMP2 zymography. TT, ZK, and TC consulted, proofread, and edited the manuscript. MS and SB developed the study concept, edited, and revised the manuscript. All authors read and approved the final manuscript.

### Conflict of Interest Statement

TT has filed and licensed patents about non-coding RNAs. TT is founder, SB is co-founder and both hold shares of Cardior Pharmaceuticals GmbH. The remaining authors declare that the research was conducted in the absence of any commercial or financial relationships that could be construed as a potential conflict of interest.

## Abbreviations

AKT: protein kinase B
AMPK: AMP-activated protein kinase
cGMP: cyclic guanosine monophosphate
ERK2: extracellular signal-regulated kinase 2
FOXO3: forkhead box O3
HFpEF: heart failure with preserved ejection fraction
Hsp: heat shock protein
IL: interleukin
LVH: left ventricular hypertrophy
Mef: myocyte enhancer factor
miR: microRNA
NO: nitric oxide
PKG: cGMP-dependent protein kinase
PTEN: phosphatase and tensin homolog
RIHD: radiation-induced heart disease
ROCK: Rho-associated kinase
TAC: transverse aortic constriction
TGF-β: transforming growth factor beta.
