# Supplementary material for: Selective Heart Irradiation Induces Cardiac Overexpression of the Pro-hypertrophic miR-212
Source: Front Oncol. 2019 Jul 16;9:598. doi: 10.3389/fonc.2019.00598 (PMC6646706; doi:10.3389/fonc.2019.00598)
Supplement: Supplementary file 1 [file Presentation_1.PPTX]

## Slide 1
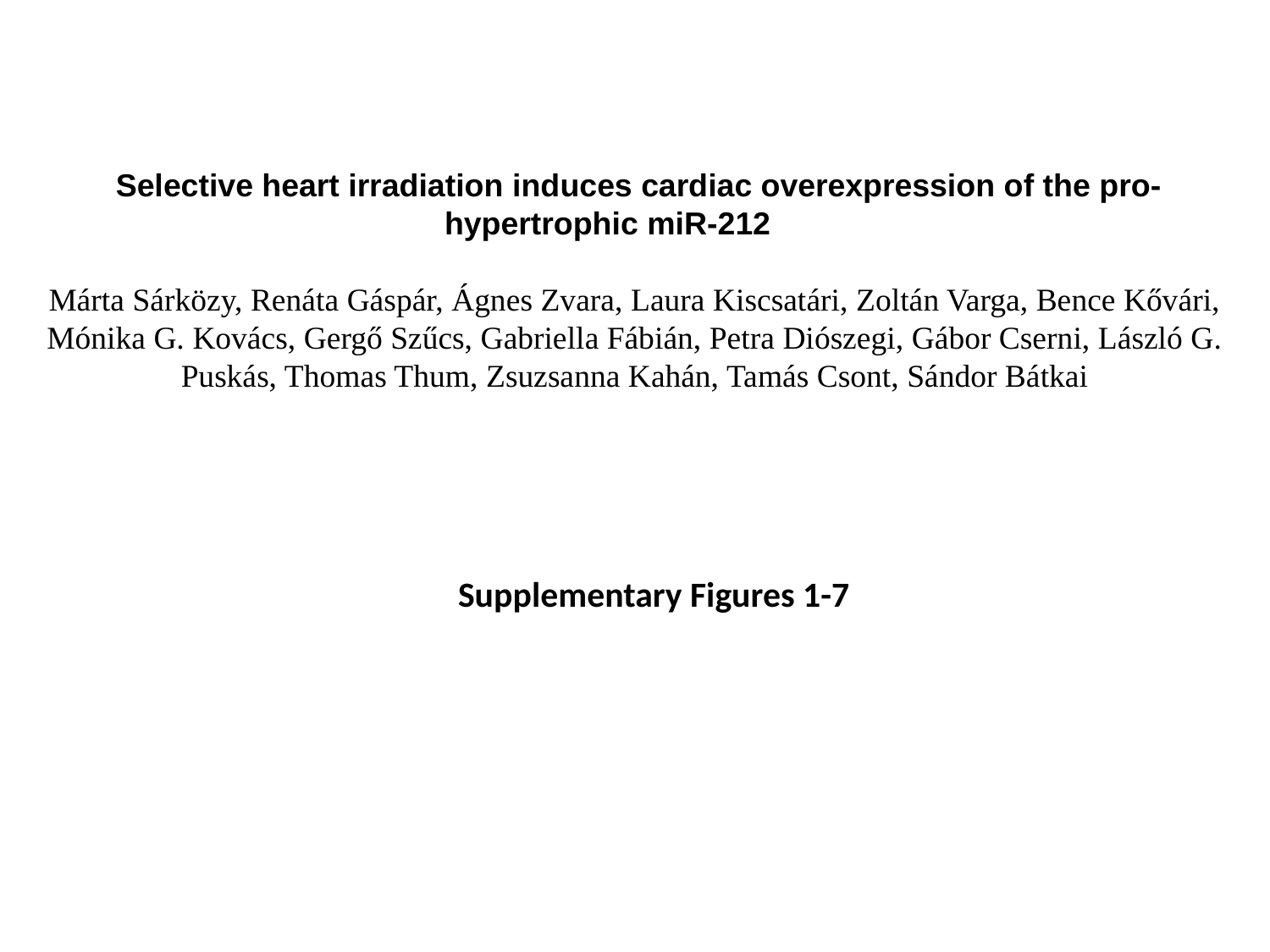

Selective heart irradiation induces cardiac overexpression of the pro-hypertrophic miR-212
Márta Sárközy, Renáta Gáspár, Ágnes Zvara, Laura Kiscsatári, Zoltán Varga, Bence Kővári, Mónika G. Kovács, Gergő Szűcs, Gabriella Fábián, Petra Diószegi, Gábor Cserni, László G. Puskás, Thomas Thum, Zsuzsanna Kahán, Tamás Csont, Sándor Bátkai
Supplementary Figures 1-7

## Slide 2
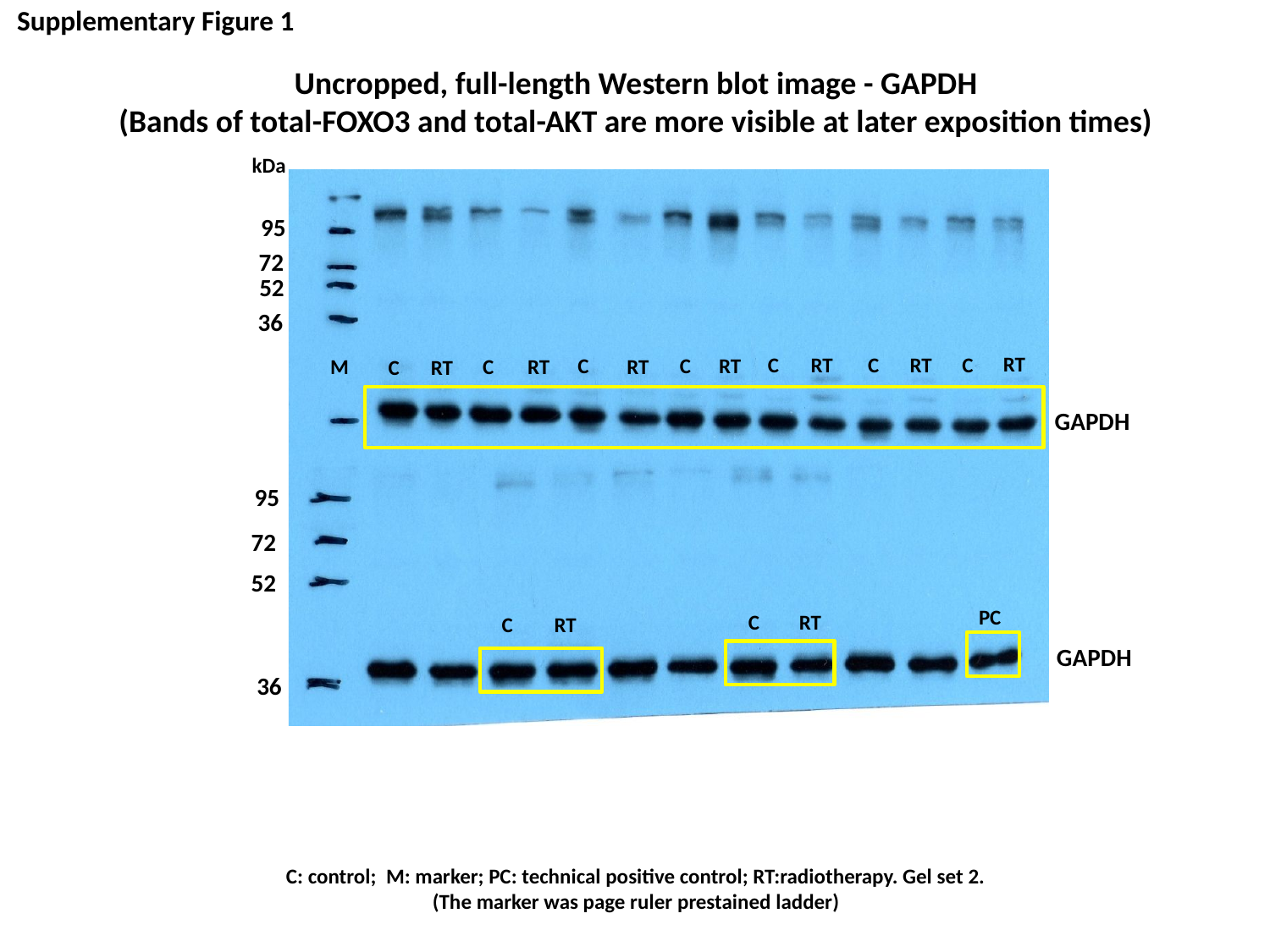

Supplementary Figure 1
Uncropped, full-length Western blot image - GAPDH
(Bands of total-FOXO3 and total-AKT are more visible at later exposition times)
kDa
95
72
52
36
RT
C
C
RT
RT
C
RT
C
C
C
RT
M
RT
C
RT
GAPDH
95
72
52
PC
C
RT
C
RT
GAPDH
36
C: control; M: marker; PC: technical positive control; RT:radiotherapy. Gel set 2.
(The marker was page ruler prestained ladder)

## Slide 3
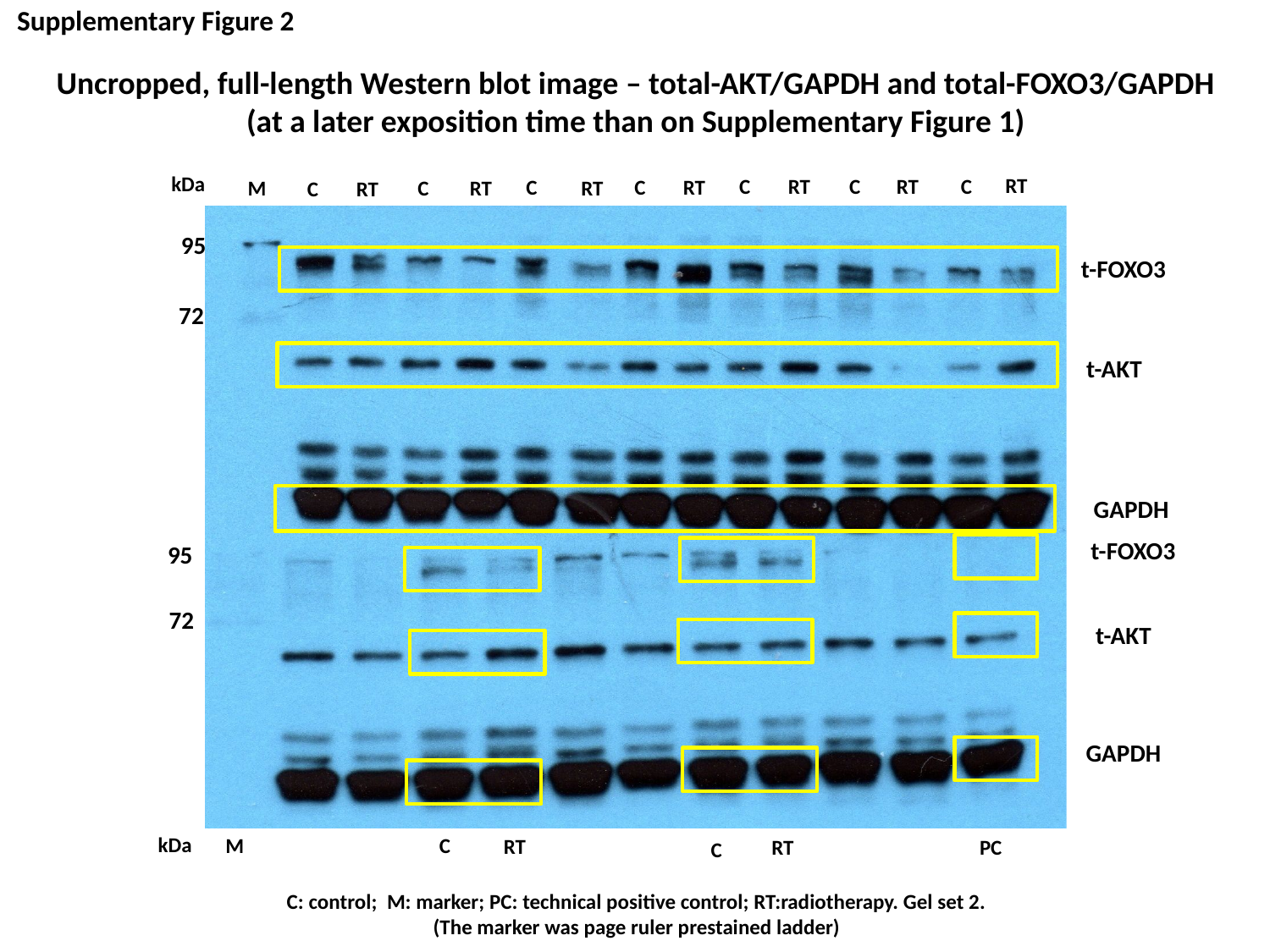

Supplementary Figure 2
Uncropped, full-length Western blot image – total-AKT/GAPDH and total-FOXO3/GAPDH
(at a later exposition time than on Supplementary Figure 1)
kDa
RT
C
C
RT
RT
C
RT
C
C
C
RT
M
RT
C
RT
95
t-FOXO3
72
t-AKT
GAPDH
t-FOXO3
95
72
t-AKT
GAPDH
kDa
M
C
RT
RT
PC
C
C: control; M: marker; PC: technical positive control; RT:radiotherapy. Gel set 2.
(The marker was page ruler prestained ladder)

## Slide 4
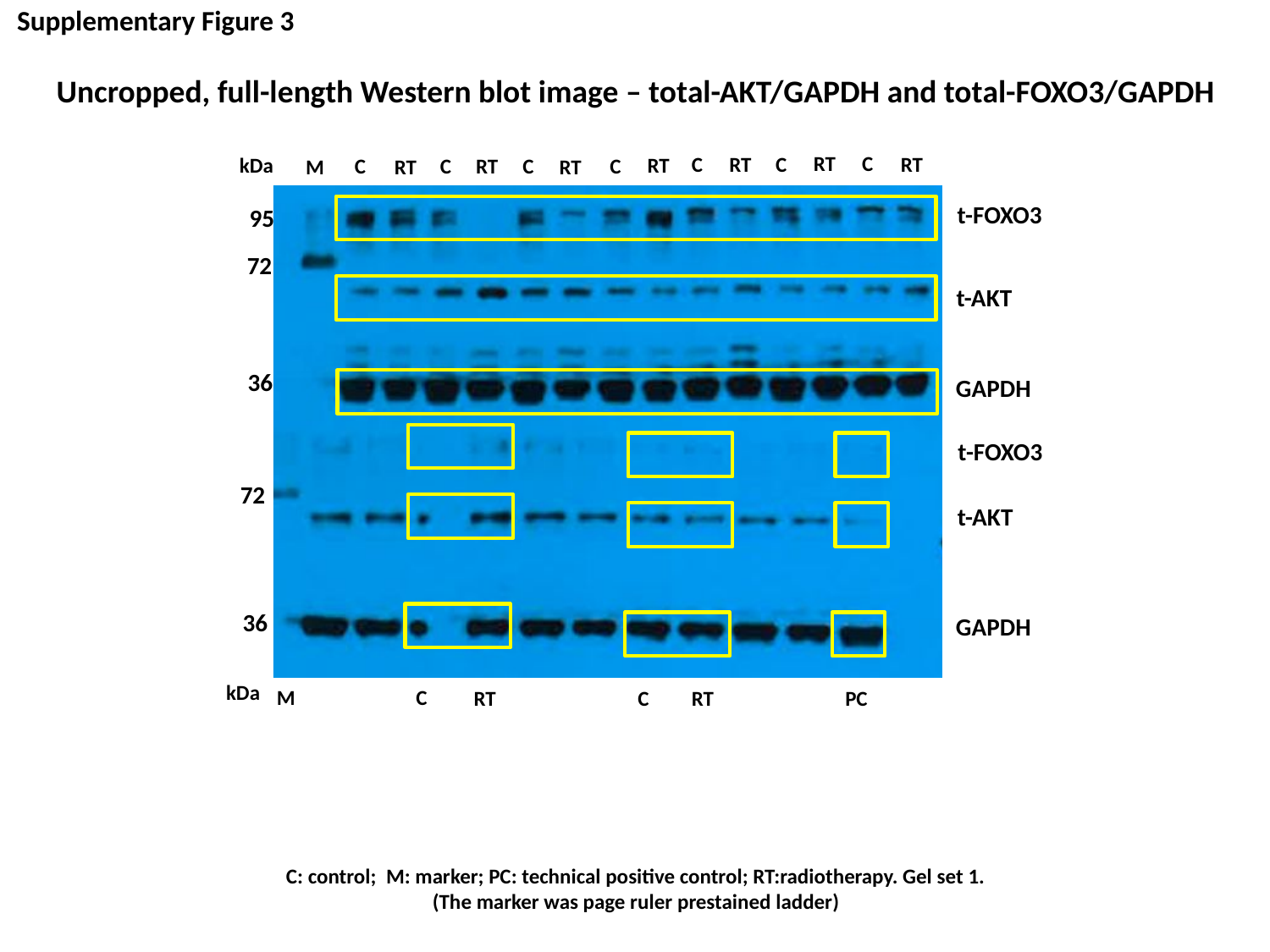

Supplementary Figure 3
Uncropped, full-length Western blot image – total-AKT/GAPDH and total-FOXO3/GAPDH
RT
C
RT
C
C
RT
kDa
RT
C
C
C
RT
C
RT
RT
M
t-FOXO3
95
72
t-AKT
36
GAPDH
t-FOXO3
72
t-AKT
36
GAPDH
kDa
M
C
RT
C
RT
PC
C: control; M: marker; PC: technical positive control; RT:radiotherapy. Gel set 1.
(The marker was page ruler prestained ladder)

## Slide 5
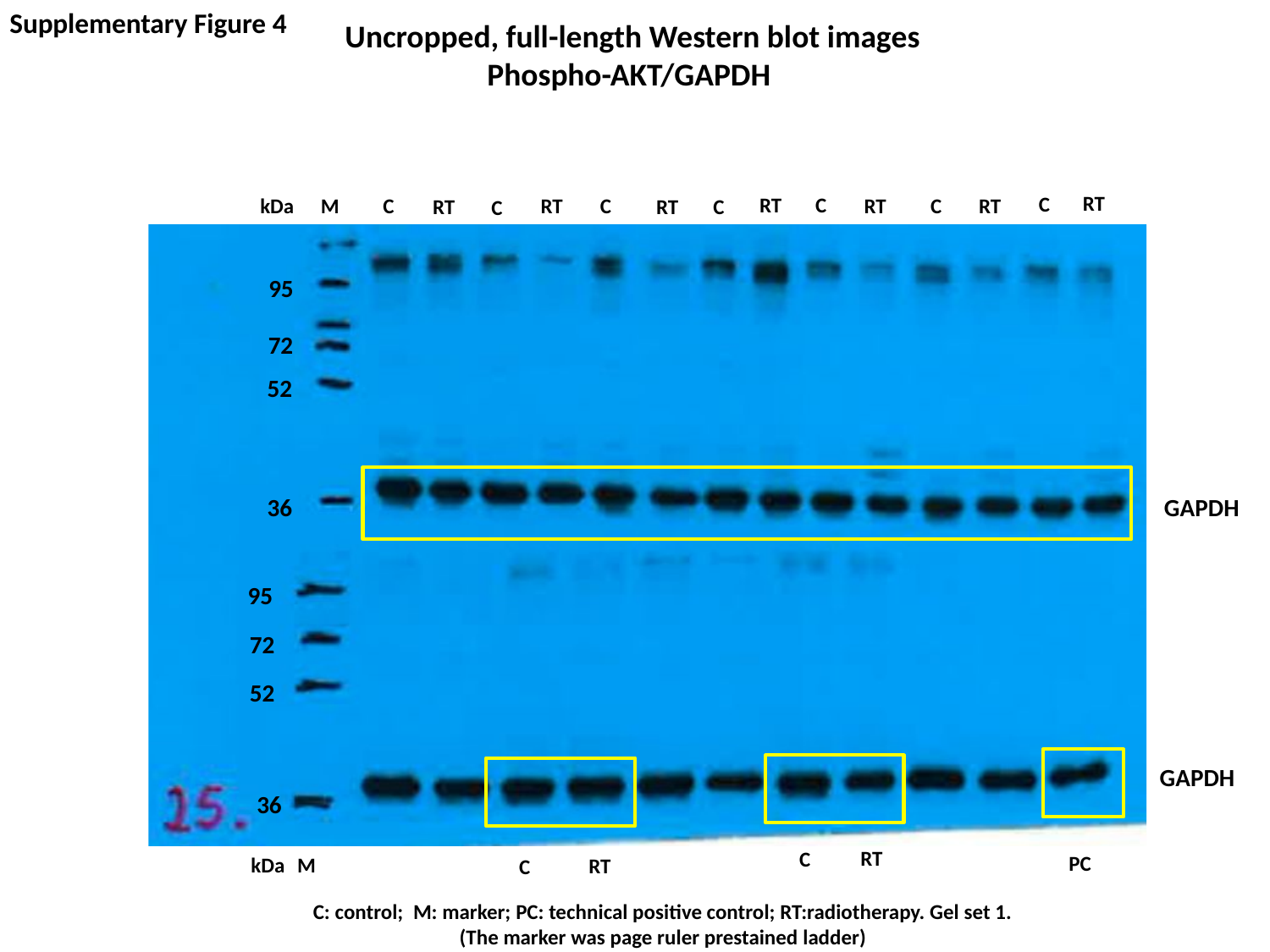

Supplementary Figure 4
Uncropped, full-length Western blot images
Phospho-AKT/GAPDH
RT
C
RT
C
RT
C
C
RT
C
M
kDa
RT
RT
C
RT
C
95
72
52
36
GAPDH
95
72
52
GAPDH
36
RT
C
PC
kDa
M
RT
C
C: control; M: marker; PC: technical positive control; RT:radiotherapy. Gel set 1.
(The marker was page ruler prestained ladder)

## Slide 6
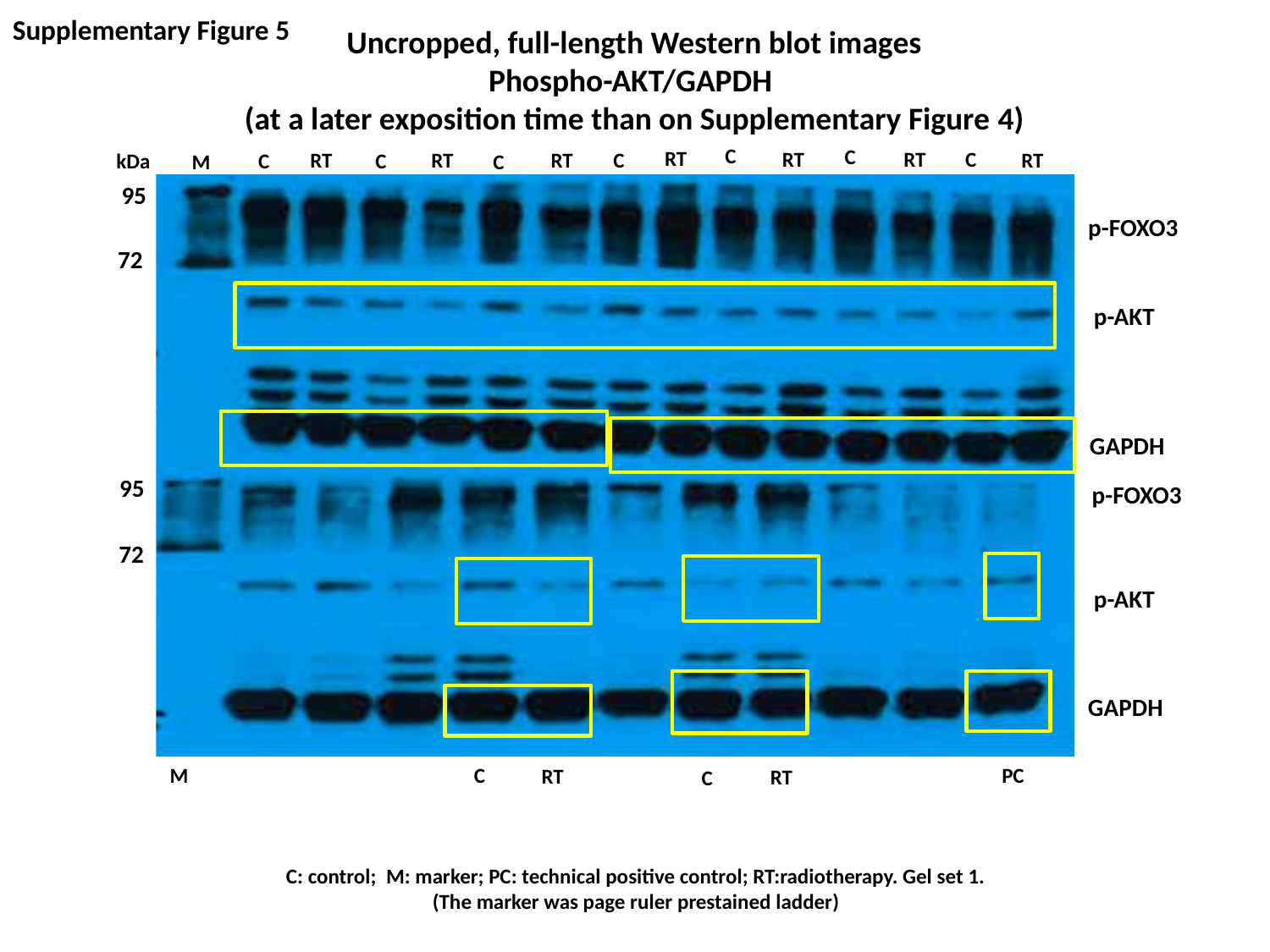

Supplementary Figure 5
Uncropped, full-length Western blot images
Phospho-AKT/GAPDH
(at a later exposition time than on Supplementary Figure 4)
C
C
RT
RT
C
RT
RT
RT
RT
RT
C
C
kDa
C
M
C
95
p-FOXO3
72
p-AKT
GAPDH
95
p-FOXO3
72
p-AKT
GAPDH
PC
M
C
RT
RT
C
C: control; M: marker; PC: technical positive control; RT:radiotherapy. Gel set 1.
(The marker was page ruler prestained ladder)

## Slide 7
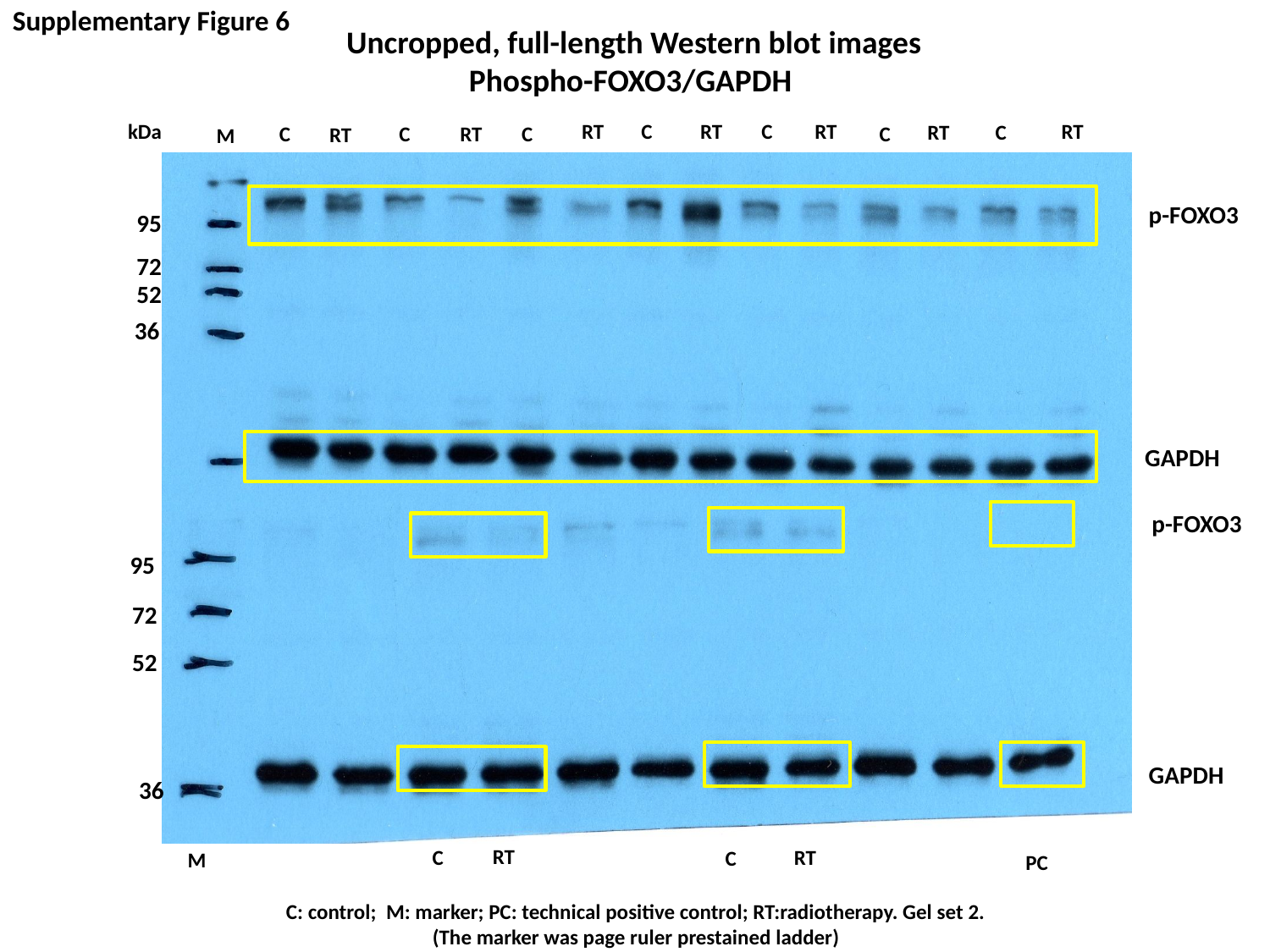

Supplementary Figure 6
Uncropped, full-length Western blot images
Phospho-FOXO3/GAPDH
kDa
RT
RT
RT
RT
C
C
C
RT
C
C
RT
C
C
RT
M
p-FOXO3
95
72
52
36
GAPDH
p-FOXO3
95
72
52
GAPDH
36
RT
C
RT
C
M
PC
C: control; M: marker; PC: technical positive control; RT:radiotherapy. Gel set 2.
(The marker was page ruler prestained ladder)

## Slide 8
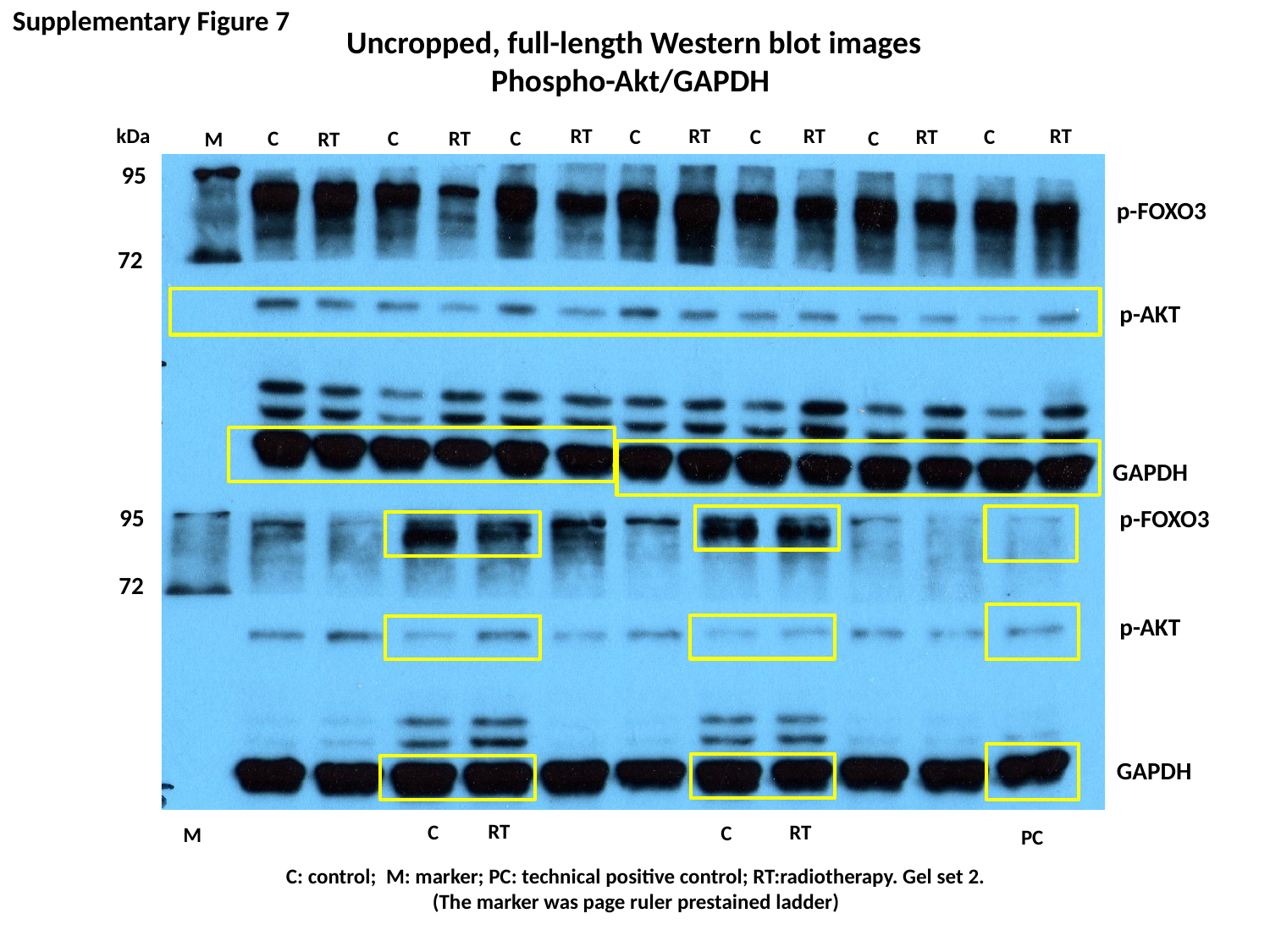

Supplementary Figure 7
Uncropped, full-length Western blot images
Phospho-Akt/GAPDH
kDa
RT
RT
RT
RT
C
C
C
RT
C
C
RT
C
C
M
RT
95
p-FOXO3
72
p-AKT
GAPDH
95
p-FOXO3
72
p-AKT
GAPDH
RT
C
RT
C
M
PC
C: control; M: marker; PC: technical positive control; RT:radiotherapy. Gel set 2.
(The marker was page ruler prestained ladder)
